# Metabolic dysfunction and insulin resistance in depression: mechanisms, evidence, and therapeutic implications

**DOI:** 10.3389/fpsyt.2026.1930167

**Published:** 2026-08-20

**Authors:** Katerina Palacek, Olivier Corbeil, Saguna Katyal, Gabriele Lo Buglio, Carl Zhou, Stanley Wong, Nicholas Fabiano

**Affiliations:** 1 Faculty of Medicine, University of Ottawa, Ottawa, ON, Canada; 2 Department of Psychiatry, University of Ottawa, Ottawa, ON, Canada; 3 Ottawa Hospital Research Institute (OHRI), Clinical Epidemiology Program, University of Ottawa, Ottawa, ON, Canada; 4 Department of Pharmacy, Quebec Mental Health University Institute, Quebec, QC, Canada; 5 University of Ottawa, School of Epidemiology and Public Health, Ottawa, ON, Canada; 6 Ottawa Hospital Research Institute (OHRI), Neuroscience Program, University of Ottawa, Ottawa, ON, Canada; 7 Department of Psychiatry, University of Toronto, Toronto, ON, Canada

**Keywords:** depression, GLP- 1 receptor agonists, glucose, insulin, MDD, major depressive disorder (MDD), metformin, mood

## Abstract

Major depressive disorder (MDD) remains one of the most frequently diagnosed mental health disorders and a leading cause of disability worldwide. The discovery of new treatments for MDD is necessary given up to 30% of individuals with MDD are resistant to current antidepressant treatments. The significant physiological interplay between MDD and metabolic conditions is crucial to consider for future therapeutics given the current obesity epidemic and the increased prevalence of treatment-resistant depression in individuals with metabolic dysfunction. The aim of this review is to discuss the relationship between metabolic dysfunction and MDD, while highlighting the potential antidepressant effects of insulin. There are interconnected perpetuating cycles involving hypothalamic-pituitary-adrenal (HPA) axis dysfunction, inflammation, obesity, insulin resistance, and MDD. Evidence on the efficacy of agents that target insulin resistance for depression is mixed and mechanisms by which these agents may improve depressive symptoms have not been clearly elucidated in studies reporting benefit. While there is a clear need to continue research on treatments that address the burden imposed by treatment-resistant depression, current data on whether insulin-sensitivity-targeting therapies can fill this gap remain limited. Clinically, routine metabolic screening in MDD could guide precision treatments targeting insulin pathways, particularly in those with metabolic dysfunction. Future studies considering the role of insulin, especially in those with insulin resistance, may enable development of novel therapeutics.

## Introduction

Major depressive disorder (MDD) is one of the most frequently diagnosed mental health disorders and a leading cause of disability worldwide (1). Almost 6% of adults suffer from depression globally (2). Symptoms of MDD include appetite changes, anhedonia, concentration and sleep difficulties, feelings of excessive guilt or worthlessness, and suicidal ideation (1). Research indicates that MDD negatively impacts multiple aspects of life, including quality of life, educational trajectories, interpersonal relationships, and work performance (1). Development of MDD depends on a variety of factors, including biological, psychological, and social (1). Current treatments for MDD include medications that target the serotonin, norepinephrine, dopamine, and glutamate systems, as well as psychotherapy, electroconvulsive therapy, transcranial magnetic stimulation, and exercise. Almost a third of individuals being treated with conventional antidepressant treatments are considered treatment-resistant (3).

Exploring the relationship between obesity, insulin, and MDD is increasingly important considering that one in eight individuals globally are obese (4). Obesity is a worldwide chronic disease that is difficult to treat, with frequent relapses and complex, multifactorial drivers (5). It is associated with metabolic dysfunction, which involves energy imbalance leading to excessive fat accumulation and disruption of normal bodily function (6). The relationship between obesity and insulin resistance is bidirectional, where obesity is a risk factor for insulin resistance, and insulin resistance can exacerbate or contribute to obesity through several interconnected mechanisms (7–10). The inflammation driven by obesity impairs the insulin signaling pathway and when this signaling pathway is impaired, more adipose tissue can be stored on the body (11). MDD, obesity, and insulin resistance are involved in increased inflammation and HPA axis dysfunction, highlighting the complexity of their relationship (12, 13). As such, the aim of this narrative review is to first discuss the interplay between metabolic dysfunction and MDD, while second highlighting the potential antidepressant effects of insulin.

## Methods

For this narrative review, we searched PubMed/MEDLINE, PsycINFO, Embase, and the Cochrane Library, supplemented by manual review of reference lists. As this was a narrative review, formal risk-of-bias assessment and GRADE (Grading of Recommendations Assessment, Development, and Evaluation) certainty ratings were not undertaken. We focused on adult clinical populations and application to children and adolescents was beyond the scope of this review.

## The role of insulin

Insulin is a polypeptide hormone that was discovered in the 1920s (14). It was the first life-saving treatment for diabetes, revolutionizing care (15). Within the same decade, the scientists involved in its discovery, Frederick Banting and John James Rickard Macleod, were awarded the Nobel Prize (15). Insulin is primarily produced and secreted by pancreatic beta cells, and its release is regulated by circulating blood glucose levels (14, 16). In the fed state, insulin lowers blood glucose through anabolic pathways, facilitating storage of circulating glucose in the liver, skeletal muscle, and adipose tissue (16, 17). To prevent hypoglycemia, several counter-regulatory hormones act in opposition to insulin, including glucagon, glucocorticoids, and catecholamines (17). Adequate insulin production, secretion, and tissue responsiveness are critical to prevent hyperglycemia. Disruption at any step leads to insulin resistance, and chronic hyperglycemia has detrimental effects on multiple organ systems.

Insulin resistance is when the body exhibits reduced sensitivity to insulin’s effects (7). This metabolic dysfunction is increasingly prevalent, particularly among younger generations as the obesity epidemic continues (18). Approximately one third of obese children and adolescents have insulin resistance (19). Insulin resistance and beta-cell failure are hallmarks of type 2 diabetes mellitus (T2DM). Initially, insulin resistance arises but cells adapt by increasing insulin secretion (17, 20). With persistent energy excess, beta cells are damaged by excess glucose and free fatty acids, ultimately resulting in uncompensated insulin resistance and T2DM (7, 17, 18, 20).

Obesity is a major contributor to insulin resistance, especially in younger populations (11). Excess adipose tissue triggers chronic low-grade inflammation in multiple tissues, causing local insulin resistance via disruption of insulin signaling pathways (11). Hepatic steatosis may further exacerbate systemic inflammation, inducing secondary insulin resistance in various tissues (11).

Clinically, insulin resistance may present as insulin resistance syndrome (17). Biochemical features include glucose intolerance, dyslipidemia, elevated procoagulant factors, hemodynamic changes, elevated inflammation markers, abnormal uric acid metabolism, and, in women, increased ovarian testosterone secretion (17). Physical signs include skin tags in individuals with obesity (21). Syndromes associated with insulin resistance include cardiovascular disease, hypertension, T2DM, metabolic dysfunction-associated steatotic liver disease (formerly known as non-alcoholic fatty liver disease), cancer, polycystic ovary syndrome, and sleep apnea (17). Insulin also exerts central effects, supporting neuronal metabolism and participating in learning and memory pathways (22). Cerebral insulin resistance can lead to neuronal degeneration and permanent memory loss and is a major risk factor for cognitive impairment (22).

Insulin resistance, diabetes, metabolic syndrome, and the aforementioned insulin-resistance-associated conditions exhibit strong comorbidity with depression (23). This raises the question of why this occurs and whether treatments for MDD should be broadened to include strategies that successfully target these other syndromes.

## The link between insulin resistance, inflammation, and MDD

**Figure 1:**
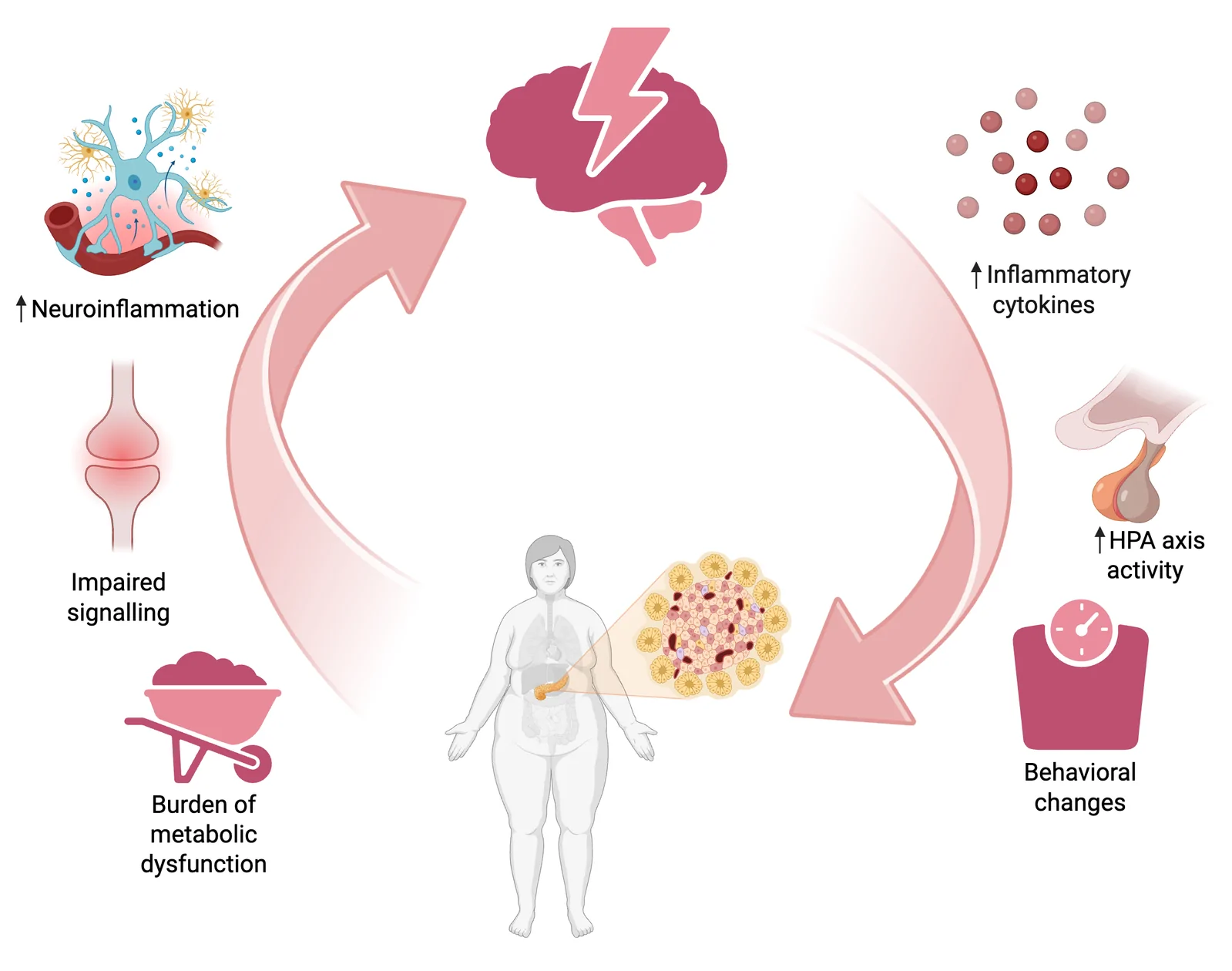
Simplified proposed perpetuating cycle linking T2DM and MDD. Created in BioRender. Palacek, K. (2026) https://BioRender.com/j515gkx.

Insulin resistance and chronic low-grade inflammation are linked, each exacerbating the other in a reinforcing feedback loop that leads to T2DM (**Figure 1**) (24). Persistent inflammation has been associated with MDD in the inflammatory hypothesis of depression. This hypothesis posits that cytokines can access the central nervous system and drive cascades that contribute to depression pathophysiology (25, 26). Cytokines can reach the brain by penetrating the blood-brain barrier or being produced locally by microglia (26). Supporting evidence includes the high comorbidity between MDD and chronic inflammatory diseases (e.g., diabetes, cardiovascular disease, autoimmune conditions) and data showing that proinflammatory cytokines can induce neurotoxicity with reduced neurotrophic support, and modulate neurotransmitter systems, amino acid metabolism, neurotrophic factors, neurogenesis, neuroendocrine activity, and mood-related neurocircuitry (25, 26). In fact, more than a third of the nonfatal burden of individuals with MDD is driven by comorbid chronic inflammatory conditions (27). Administration of cytokines can generate depressive symptoms, and several approved MDD treatments demonstrate anti-inflammatory effects (26). Proinflammatory cytokines can also impair central negative feedback regulation of the hypothalamic-pituitary-adrenal (HPA) axis and contribute to glucocorticoid resistance (26). The HPA axis coordinates responses to stressors threatening homeostasis; cytokines are among the signals it integrates (28). Chronic imbalance of these cytokines may therefore dysregulate the HPA axis (28).

Chronically elevated inflammatory cytokines in obesity and depression can signal persistent stress to the HPA axis, promoting its overactivity. Sustained HPA activation favors visceral adiposity, creating a vicious cycle (29). Glucocorticoids decrease insulin secretion, increase insulin resistance, and promote abdominal fat, further perpetuating the cycle (29). Cortisol, the primary glucocorticoid, can accelerate insulin resistance by reducing insulin sensitivity, promoting gluconeogenesis, and increasing abdominal adiposity (29, 30).

HPA axis dysfunction is strongly correlated with MDD. Some studies suggest that alterations in HPA functioning are crucial to the pathogenesis of depression (31–33), potentially via interactions with inflammatory pathways (34). MDD is frequently associated with chronic stress, which can induce HPA hyperactivity, creating a bidirectional cycle wherein depression and HPA axis dysfunction reinforce one another (35). Thus, perpetuating cycles link HPA axis dysfunction, obesity, insulin resistance, and MDD.

Obesity is a major risk factor for T2DM (32, 36). Risk is particularly elevated with intra-abdominal adiposity (37). Obesity can drive T2DM by inducing insulin resistance and beta-cell dysfunction through adipose tissue fibrosis, inflammation, neural alterations, intracellular disturbances, and altered intercellular signaling that reduces tissue insulin sensitivity (37–40). Weight loss can restore metabolic function when sufficient beta-cell recovery is possible (37).

Epidemiological data indicate that insulin resistance and MDD are strongly connected (41). Approximately one in four individuals with T2DM has either MDD or subthreshold depression (42, 43). While such a relationship is well supported by epidemiological evidence, the mechanisms linking insulin resistance and depression, as well as the directionality of such a relationship, require further research (44).

## The potential of insulin as an antidepressant

Conceptually, current antidepressants primarily target dysregulated neurotransmitters (e.g., serotonin, norepinephrine, dopamine). Although insulin is a peripheral metabolic hormone, insulin receptors are also found in brain regions involved in mood regulation and neurotransmitter production, including the raphe nuclei, amygdala, ventral tegmental area, and nucleus accumbens (45). In mice, Gupta et al. found that insulin increased brain serotonin levels and produced antidepressant effects (46). Rodent studies further indicate that insulin resistance impairs dopamine signaling and generates depressive and anxious phenotypes (47). Cerebral insulin resistance can also impair neurogenesis and synaptic plasticity within reward and learning pathways, potentially contributing to depressive symptoms (45, 48). In rats, Mueller et al. reported that insulin achieved antidepressant-like effects comparable to a serotoninergic antidepressant, mediated via insulin-like growth factor-1 (IGF-1) receptors (49). Notably, several studies suggest that selective serotonin reuptake inhibitors (SSRI) and ketamine may enhance IGF-1 signaling, potentially linking insulin pathways to conventional antidepressant mechanisms (50–52).

Insulin resistance has been linked to depression that is non-responsive to conventional antidepressants (53). This has been proposed to reflect persistent neurotransmitter dysregulation secondary to insulin resistance (53, 54). Therefore, in individuals with insulin resistance, conventional antidepressants may only partially restore neurotransmitter function if insulin signaling remains impaired.

While insulin appears to influence neurotransmitter systems long considered central to depression, some conventional antidepressants may increase insulin sensitivity and have positive metabolic effects (55). These effects have been most consistently observed with SSRIs, whereas drugs that act more strongly on the norepinephrine system have been associated with reduced insulin sensitivity (55). Consistent with this, in the GENome-based therapeutic Drugs for DEPression (GENDEP) project, obesity predicted a poorer response to nortriptyline (noradrenergic/serotonergic) but not to escitalopram (principally serotonergic) (56).

Taken together, targeting insulin resistance and metabolic dysfunction may be a viable strategy for treating depression. Given the high rates of non-response to conventional therapies, identifying new targets is a priority (45). This is particularly relevant because many individuals with treatment-resistant depression exhibit metabolic dysfunction, including insulin resistance (57, 58). Considering the role of insulin, especially in those with insulin resistance, may therefore enable novel treatments and improved outcomes.

## The bidirectional relationship and interpretation of treatment effects

The relationship between insulin resistance and MDD is bidirectional. A meta-analysis including 70 studies (n=240,704) found that fasting insulin levels and HOMA-IR were significantly elevated during acute depressive episodes, but not during remission, demonstrating that insulin resistance functions as a state rather than trait biomarker (59). Mendelian randomization analyses further support bidirectional causal effects, with genetic risk of MDD associated with increased risk of T2DM and markers of insulin resistance, while genetic risk of T2DM is associated with MDD and even treatment-resistant depression (60). While treatments that target and improve metabolic dysfunction and insulin sensitivity have been associated with significant mood improvements in some trials (61–63), it is important to note that changes in metabolic markers do not always correlate with the antidepressant effects of insulin-sensitizing agents (64). As such, some of the observed improvements in MDD symptoms following insulin-sensitizing agents may reflect non-specific benefits such as enhanced physical well-being or increased energy rather than a specific isolated antidepressant mechanism mediated by insulin pathways. These considerations are crucial to delineate causality in future trials via mediation analyses to better distinguish the insulin-specific versus non-specific antidepressant effects.

## Treatments that target insulin signaling

While a systematic review of 28 studies found insulin resistance to be associated with depressive symptom severity and found individuals with depression to have higher fasting glucose (SMD = 0.30, 95% CI [0.12, 0.48]) (65), evidence on the efficacy of anti-hyperglycemic agents for depressive symptoms is mixed (41). Furthermore, the mechanisms by which these agents might alleviate depressive symptoms are not fully elucidated (41). It remains unclear whether mood benefits (when observed) are mediated by improved insulin sensitivity per se, secondary anti-inflammatory or neurotrophic effects, weight loss, or other pathways (41). The following section will describe some of the current available treatments for metabolic dysfunction and insulin resistance, while highlighting evidence that discusses their therapeutic implications with depression. While this section does not provide a comprehensive overview, we aim to broadly highlight the potential of these agents. These treatments include pharmacological management with exogenous insulin, metformin, peroxisome proliferator-activated receptor-gamma (PPARγ) agonists, glucagon-like peptide-1 (GLP-1) receptor agonists, and sulfonylureas, as well as lifestyle measures such as exercise and dietary changes.

One approach to augment insulin signaling is exogenous insulin. Even when tissue responsiveness is diminished, additional insulin may potentiate signaling. In the literature featuring patients with and without diabetes reviewed by Woo et al., mixed evidence was found regarding the impact of insulin administration on depressive symptoms (41). One study analyzing poorly controlled type-2 diabetic patients who did not all have depressive symptoms at baseline reported improvements in depressive symptoms at 4 and 12 weeks when on a median daily insulin dose of 32U (41, 66). In a follow-up study with the same patient population demographics as the one previous, the improvement in depressive symptomology was not observed on the longer time scale of 6 months when targeting a capillary blood glucose of <7mmol/L fasted and <10mmol/L before meals and bedtime (41, 67).

Metformin improves insulin sensitivity primarily by activating the AMPK signaling pathway, but also by acting through other methods (68). It is widely used in the treatment of T2DM (68, 69). In a randomized-controlled study of 58 patients with MDD and T2DM, 24 weeks of metformin significantly improved depressive symptoms (MADRS and HRDS-17) and cognitive performance, compared to the placebo group (61). In a cohort of 323,930 people with T2DM, metformin use was associated with a significantly lower risk of depression (70). However, a meta-analysis of 6 randomized controlled trials, comprising of 2,638 T2DM individuals, did not find consistent benefit on depressive symptoms (71). This being said, most studies in this meta-analysis were not focused on patients with MDD (71).

PPARγ agonists treat insulin resistance by modulating glucoregulatory pathways (72). Evidence suggests promising antidepressant effects (62). A meta-analysis by Colle et al. found that 30 mg per day of PPARγ agonists could induce remission of depressive symptoms in individuals with depression, including those with and without metabolic comorbidities (62). Three of the studies included in this meta-analysis had a study duration of 6 weeks, and the fourth had a study duration of 12 weeks (62). In the meta-analysis by Moulton et al., pioglitazone, a PPARγ agonist, was associated with improvement in depressive symptoms when compared to controls with a dose range of 15 to 45 mg per day between studies which ranged in duration from 6 to 44 weeks (pooled effect size = -0.68) (71).

GLP-1 receptor agonists, popularized for weight loss, also improve insulin resistance by increasing glucose transporters in tissues and reducing inflammation (73). While evidence for reducing incident depression in diabetes is mixed, a meta-analysis by Chen et al. found that GLP-1 agonists can significantly reduce depressive symptoms, compared to control treatments (SMD = -0.12, 95% CI [-0.21, -0.03], I^2^ = 2%) (63, 74).

Sulfonylureas have been observed to increase pancreatic insulin secretion and reduce hepatic gluconeogenesis and insulin clearance (58). In a cross-sectional study, exposure to sulfonylureas was associated with lower odds of depression (PHQ-9) in patients with T2DM (OR of 0.374, 95% CI: 0.182–0.768, P = 0.007)\ (75).

Finally, exercise and dietary changes are long known to increase insulin sensitivity (76). In a systematic review and meta-analysis accounting for 2264 adults with a diagnosis of MDD or depressive symptoms, exercise was shown to have moderate to large positive effects on depressive symptoms (77). This effect remained even when limiting the analysis to low risk of bias studies (77). Furthermore, non-inferiority trials indicated that exercise was not inferior to current first-line antidepressants (77). While weight loss shows mixed effects on mood overall, among overweight individuals, caloric restriction has been associated with improved depressive symptoms in limited evidence (78).

## Discussion

Evidently, the evidence surrounding insulin and drugs that target insulin resistance is inconclusive. Nonetheless, the mixed results highlighted above illustrate the importance of further delineating the effects of these potential therapeutics with larger randomized control trials (RCTs). The limited analysis in this review is primarily due to a paucity of literature but exacerbated by high interstudy heterogeneity (74). Furthermore, a lot of the evidence comes from preclinical studies, observational data and small clinical trials. Finally, many of the studies that analyze if the aforementioned treatments can improve depressive symptoms are performed in patients without diagnosed depression.

If future testing with larger RCTs demonstrate null effects, this could indicate that the theory surrounding insulin and insulin resistance in depression discussed above requires refinement. Notably, this may suggest that these agents would be better suited as adjuncts than as an independent therapy. Furthermore, the patient population in which these agents would be best suited may require further studies to delineate, as it is possible that these agents are effective in the treatment of depressive symptoms in individuals with metabolic dysfunction, but less so in individuals without. Finally, it would also be of relevance to parse out how these agents affect conditions related to MDD including sleep and cognitive disorders, and how its effect on these conditions affect the prognosis of depression. More targeted work surrounding potential therapeutics for depression must take into consideration the multifactorial onset of this disorder where one treatment option may not target all cases of depressive symptom pathology.

MDD remains highly prevalent and disabling, and non-response to conventional antidepressants underscores the need for novel strategies. Robust physiological links between MDD and metabolic dysfunction, particularly insulin resistance, highlight a plausible therapeutic axis. Although early data suggest that insulin-sensitivity-targeting interventions may alleviate depressive symptoms in some patients, the overall evidence is mixed, and the underlying mechanisms are not fully delineated. Clinically, routine metabolic screening in MDD could inform precision-medicine approaches in which insulin pathways are considered as adjunctive targets, especially for those with clear metabolic dysfunction. Insulin-targeted therapies have transformed the management of metabolic disease; appropriately tested and judiciously applied, they may also contribute to reducing the burden of MDD.
